# Sirtuin1, 3 and 6 in serum of Alzheimer's Disease patients: An approach to establish as non‐invasive biomarkers

**DOI:** 10.1002/alz70856_106305

**Published:** 2026-01-09

**Authors:** Abhinay Kumar Singh, Sharmistha Dey

**Affiliations:** ^1^ All India Institute of Medical Sciences, New Delhi, Delhi, India; ^2^ All India Institute of Medical Sciences, New Delhi, CA, India

## Abstract

**Background:**

Alzheimer's disease (AD) is the most prevalent neurodegenerative disorder. Dysfunction of mitochondria and oxidative stress are known to aggravate the disease pathology. Sirtuins, NAD‐dependent deacetylases, have a well‐defined role in this pathway and thus can serve as a potential biomarker for the early detection of the disease.

**Method:**

This study evaluated the level of serum Sirtuins (SIRT1, SIRT3 and SIRT6) in three study groups: AD, mild cognitive impairment (MCI) and geriatric control (GC) by the label free surface plasmon resonance (SPR) technology and was further validated by the Western blot experiment. ROC analysis was performed to differentiate the study group based on the concentration of serum SIRT proteins.

**Result:**

The serum level of SIRT1, SIRT3 and SIRT6 (mean ± SD) were significantly decreased in AD (1.65 ± 0.56, 3.15 ± 0.28, 3.36 ± 0.32 ng/μl), compared to MCI (2.17 ± 0.39, 3.60 ± 0.51, 3.73 ± 0.48 ng/μl) and GC (2.84 ± 0.47, 4.55 ± 0.48, 4.65 ± 0.55 ng/μl). ROC analysis showed the cut‐off value with high sensitivity and specificity for cognitive impairment (AD and MCI). The concentration declined significantly with the disease progression.

**Conclusion:**

This study for the first time reports the concentration of SIRT1, SIRT3 and SIRT6 in serum of AD and MCI patients and reveals an inverse relationship of serum level of SIRT1, SIRT3 and SIRT6 with AD. The cut‐off values with sensitivity and specificity shows the clinical relevance of SIRT3 and SIRT6 as serum protein markers for AD.

